# Assessing the needs of grandparents of preterm infants in neonatal intensive care units: a cross-sectional study

**DOI:** 10.3389/fpsyg.2024.1433391

**Published:** 2024-11-19

**Authors:** Huamin Huang, Jingyun Tao, Ying Lei, Rui Chen, Haixia Fang

**Affiliations:** ^1^Pediatric Department, the Second People’s Hospital of Jingdezhen, Jingdezhen, Jiangxi, China; ^2^Emergency Department, the Second People’s Hospital of Jingdezhen, Jingdezhen, Jiangxi, China

**Keywords:** preterm infants, neonatal intensive care unit, grandparents, critical illness, family needs, China

## Abstract

**Background:**

Globally, there is an increasing trend in the incidence of premature births and low birth weight. Neonatal intensive care unit (NICU) care has become indispensable for these newborns. Nevertheless, this mode of care poses substantial economic, psychological, and health challenges to the families of preterm infants. Despite abundant evidence concerning the parents’ needs in the NICU, the needs of grandparents—vital family members—are frequently disregarded. This exploratory study aimed to assess the grandparents’ needs of preterm infants in the NICU, exploring the impact of demographic elements on these needs to offer guidance for clinical care practices.

**Methods:**

This study employed a cross-sectional design and the Chinese version of the NICU Family Needs Inventory (NICU-FNI) to investigate the needs of grandparents. A total of 280 grandparents participated in the study, providing data by completing structured questionnaires related to their demographic profiles and needs. Statistical analyses were utilized to analyze the data, including descriptive statistics, chi-square tests, Pearson’s correlation, and multiple linear regression.

**Results:**

Six items about Assurance emerged as significant among the top 10 important needs, with two items for Information, one for Proximity, and one for Support; among the least important needs, nine items related to Comfort and Support were identified. The subscale “Assurance” achieved the highest mean score of 4.07 ± 0.49, followed by the subscales of “Information” and “Proximity,” registering mean scores of 3.50 ± 0.47 and 3.50 ± 0.46, respectively. This explorative study identified a correlation between the needs for Assurance and employment status, place of residence, gestational age, and birth weight (*p* < 0.05). Employment status, place of residence, and gestational age were identified as significant correlates for Assurance (*p* < 0.05).

**Conclusion:**

The foremost need identified by grandparents is Assurance of quality care for preterm infants, closely followed by the demand for thorough Information and the ability to be in Proximity to the infant. This exploratory study highlights that mitigating the strain on families with preterm infants, as well as recognizing and meeting the needs of grandparents, is of paramount importance.

## Introduction

The neonatal intensive care unit (NICU) offers life-saving care; however, the exorbitant expenses and intricate caregiving procedures can impose considerable emotional and psychological burdens on the entire family, precipitating conditions such as depression, anxiety, and trauma (Geng et al., 2022; Grunberg et al., 2022). Generally, neonates necessitating urgent medical intervention are admitted to the NICU (Chow et al., 2015). Such infants are commonly characterized by preterm birth (i.e., occurring prior to 37 weeks of gestation), low birth weight, and/or the presence of severe medical ailments (Chow et al., 2015). As per the WHO’s “Global Report on Preterm Births,” the worldwide occurrence rate of preterm or low birth weight deliveries increased from 9.6% in 2005 to 11.1% in 2012 (WHO, 2012). Furthermore, in a 2011 nationwide survey in China, the preterm birth incidence was recorded at 7.1% (Zou et al., 2014). Following the relaxation of China’s two-child policy in 2015, the surge in advanced maternal age, multiparous women, and multiple gestation pregnancies has significantly led to an escalation in the preterm birth rate (Yang et al., 2017; Zhang et al., 2021).

Parents of premature infants are frequently confronted with a variety of challenges pertaining to economic, psychological, and physical health. Upon the admission of their infants to the NICU, these parents often undergo emotions of anxiety, depression, guilt, and a sense of helplessness, potentially adversely (Kim, 2018; Lorié et al., 2021). Identifying and addressing parental needs in the NICU is a crucial factor in achieving the best outcomes for newborns and their families. Specifically, parents of preterm infants have emphasized the significance of obtaining infants’ health information and guidance (Arockiasamy et al., 2008; Harris et al., 2024), trusting medical staff (Bernaix et al., 2006), and benefiting from Support from staff to meet their needs (Bernaix et al., 2006; Pepper et al., 2012).

The emergence and evolution of the concept of “family-centered care” has led to active parental involvement as primary caregivers in the care of preterm infants, representing critical factor in enhancing the post-discharge survival quality of preterm infants; the majority of research on the family needs in NICU has mainly concentrated on newborn parents (Arockiasamy et al., 2008; Diffin et al., 2013), particularly on mothers of preterm infants (Govindaswamy et al., 2020). Usually, mothers may suffer physical discomfort after delivery, making it impossible to care for the baby; fathers serve as the initial point of contact between the family and the NICU staff and are the ones who decide on the need for emergency treatment (Arockiasamy et al., 2008; Johnson, 2008). Nevertheless, grandparents play a pivotal role in the family, especially for parents of preterm infants in single-child households. In China, they hold an especially significant position, often enjoying relatively high economic status and social standing (Zhang et al., 2022). Moreover, there is a high prevalence of grandparents living with their grandchildren in China. These resident grandparents play a vital role in raising the children, particularly once the child has passed their first birthday. While caring for the children, grandparents often work with healthcare professionals to ensure the children’s wellbeing. This includes regular health checkups, vaccinations, and monitoring of the children’s development, requiring the active involvement and cooperation of the grandparents (Chen et al., 2011; Chen and Lewis, 2015). Chinese fathers often rely on grandparents for support to reduce their stress, considering the mother’s need for care. Grandparents are viewed as the most trusted individuals (Sun et al., 2024).

Studies have demonstrated that, in the context of a family member’s acute severe illness, the concept of family resilience is instrumental in facilitating adaptive responses and preserving familial equilibrium in the face of both internal and external crises (Gao et al., 2022; Shao et al., 2023; Walsh, 2016). The critical role of grandparents in enhancing family resilience is unmistakable; they provide support and comfort to younger parents, thereby strengthening the family’s collective resilience through shared confrontations with adversities (Lambiase et al., 2024). In the high-pressure environment of the NICU, grandparental support helps alleviate the emotional strain on parents, increases familial cohesion, and enhances the family’s ability to navigate hardships (Brødsgaard et al., 2017). Hence, grasping the needs of grandparents with a premature infant in the NICU is of utmost importance; healthcare professionals should be adequately prepared to meet grandparents’ needs by offering the necessary support and assistance. Such proactive measures can significantly reduce the grandparents’ emotional burden, by extension, on the parents, establishing a constructive dialog between patient families and healthcare providers, thereby reducing the incidence of medical disputes (Brødsgaard et al., 2017; Jain et al., 2021; Sun et al., 2024). This exploratory study aims to investigate the unique needs of grandparents with preterm infants in the NICU. The primary aim is to generate initial insights that will assist healthcare professionals in understanding how to provide more tailored and effective support and to offer foundational information to regulatory authorities as a basis for potential considerations in developing NICU policies.

## Materials and methods

### Study design and setting

The NICU-Family Need Inventory (NICU-FNI) is a specialized adaptation of the Critical Care Family Needs Inventory (CCFNI) by Ward (2001), incorporating the research by Jacono et al. (1990) and Kirschbaum (1990), explicitly designed to address the needs of parents with NICU infants. The NICU-FNI is divided into five domains (Leske, 1991; Ward, 2001): Support (interpersonal and emotional needs); Comfort (personal and physical comfort); Information (pragmatic information about the infant); Proximity (remain in the range of infant); and Assurance (experience confidence about the infant). The NICU Family Needs Inventory comprised 56 items to measure the importance attributed to parents’ needs using a 5-point Likert scale incorporated into each item: 5 = extremely important, 4 = important, 3 = moderately important, 2 = unimportant, and 1 = extremely unimportant. Zhang et al. (2016) translated the NICU-FNI scale into Chinese and evaluated the reliability and validity of the scale, with results indicating Cronbach’s alpha coefficient of 0.957 for the translated NICU-FNI scale, a test–retest reliability of 0.875, and a content validity index (CVI) of 0.934. The correlation coefficients between each dimension and the total score ranged from 0.753 to 0.872, thus demonstrating good reliability and validity in the preliminary testing of the Chinese version of the NICU-FNI scale among parents with a premature infant in the NICU in China. In this study, we utilized the Chinese version of the NICU-FNI scale (Zhang et al., 2016) and re-evaluated its reliability and validity. The findings indicated Cronbach’s alpha coefficient of 0.963 and a test–retest reliability of 0.792 for the NICU-FNI scale, demonstrating good reliability and validity in preliminary testing among grandparents of premature infants in the NICU in China. Building on previous studies (Ward, 2001; Zhang et al., 2016), this questionnaire (see Supplementary material) was developed in two parts: the first part collected demographic information on grandparents, mothers, and newborns, while the second part consisted of the Chinese version of the NICU-FNI scale (40 items).

### Study population and sample size

The cross-sectional study was conducted at the Obstetrics and Gynecology office of the Second People’s Hospital of Jingdezhen between February and December 2023. Stringent selection procedures identified a cohort of grandparents visiting infants hospitalized in the NICU as the study sample. In order to uphold the strictness and precision of the research, obstetric nurses were tasked with issuing research invitations to them and delivering comprehensive research information sheets in print format. Upon reception of the information sheets, these grandparents were asked to read and comprehend the contents diligently. Following this, we instructed the grandparents who agreed to participate in the research to complete a questionnaire survey. The inclusion criteria for this study were grandparents whose premature grandchildren were still receiving treatment in the NICU at the time of questionnaire completion. Using the mother’s admission number for preterm infants or the infants’ own admission numbers as unique identifiers, one grandparent was chosen to participate in the survey. In cases involving twins, two grandparents were chosen for the study. Exclusion criteria included grandparents of infants whose grandchildren had completed NICU treatment and were discharged and grandparents who did not understand Chinese.

We calculated the sample size using the Power Analysis and Sample Size (PASS) Version 15.0 software (NCSS, LLC, Kaysville, Utah, USA). A sample of 258 participants, each responding to 40 items, achieves 90% power to detect the difference between the coefficient alpha under the null hypothesis of 0.80 and the coefficient alpha under the alternative hypothesis of 0.85. This is accomplished using a two-sided *F*-test with a significance level of 0.05 (Bonett, 2002). Considering the estimated 10% non-response rate, we calculated the expected sample size to be 287 participants.

Ethical clearance for this research was granted by the Ethics Committee of the Second People’s Hospital of Jingdezhen, documented by Approval No: 2023-LLLW-07. Moreover, it conformed to the Declaration of Helsinki principles, guaranteeing that informed consent was obtained from all participants.

### Statistical analysis

Utilizing descriptive statistics, the study summarized survey responses, representing data in tables with counts and percentages for response categories, assessing response frequency differences across groups using a chi-square test, conducting a one-way ANOVA for comparisons among three or more groups, and investigating correlations between dependent and independent variables through Pearson’s correlation analysis. Univariate linear regression and multivariate multiple linear regression analyses were conducted to find factors relevant to identifying grandparents’ needs. All statistical analyses were performed using SPSS statistical software (version SPSS 20.0, IBM SPSS Inc., Chicago, IL, USA). *p-*values <0.05 were considered to indicate statistical significance.

## Results

### Demographics of the respondents

The questionnaires were distributed to 287 grandparents, with 280 completed and returned. All respondents belonged to independent families associated with the NICU, meaning that each family had only one grandparent complete the questionnaire, resulting in a response rate of 97.6%. As shown in Table 1, among these 280 participants, grandmothers constituted 50.7%. The mean age of participants was 59.6 years (S.D. = 7.2 years). Regarding age groups, the proportions were as follows: ≤55 years (26.4%), 56–60 years (30.4%), 61–65 years (23.2%), and > 65 years (20.0%). In terms of place of residence, 45.0% of the grandparents lived in towns or cities, whereas 55.0% resided in the countryside. In terms of education level, the proportions were as follows: college and above (18.6%), junior high school/technical secondary school (27.5%), junior middle school (26.8%), and primary school and below (27.1%). Furthermore, employment status distribution includes full-time employment (23.6%), part-time employment (22.1%), unemployment (26.8%), and retirement (27.5%).

**Table 1:** Demographics of grandparents (*n* = 280).

Characteristics	Frequency (*n*, %)	*p*-value
Sex		
Male	138 (49.3)	0.811
Female	142 (50.7)	
Age group (years)		
≤55	74 (26.4)	0.086
56–60	85 (30.4)	
61–65	65 (23.2)	
>65	56 (20.0)	
Place of residence		
Town/city	126 (45.0)	0.094
Countryside	154 (55.0)	
Education level		
College and above	52 (18.6)	0.102
Junior high school/technical secondary school	77 (27.5)	
Junior middle school	75 (26.8)	
Primary school and below	76 (27.1)	
Employment status		
Employed full-time	66 (23.6)	0.532
Employed part-time	62 (22.1)	
Unemployed	75 (26.8)	
Retired	77 (27.5)	

All calculations were performed using the one-way ANOVA, except for sex and place of residence, which were evaluated using the chi-square tests.

As shown in Table 2, the distribution of newborn characteristics revealed significant variations in gestational age, birth weight, and admission diagnosis. Gestational age categories were as follows: 28–34 weeks (9.3%), 34–37 weeks (27.9%), and over 37 weeks (62.9%), showing a statistically significant difference (*p* < 0.001). Birth weight categories included under 1,500 g (1.4%), 1,501–2500 g (34.3%), and over 2,501 g (64.3%), with a notable statistical significance (*p* < 0.001). In terms of admission diagnosis, the frequencies were as follows: neonatal jaundice (37.9%), neonatal infectious pneumonia (36.4%), neonatal hypoxic–ischemic encephalopathy (10.7%), premature infants (8.6%), neonatal respiratory distress syndrome (5.0%), and neonatal asphyxia (1.4%). The differences in admission diagnoses were also found to be statistically significant (*p* < 0.001).

**Table 2:** Characteristics of the preterm infants in neonatal intensive care units (*n* = 280).

Newborn characteristics	Frequency, *n* (%)	*p*-value
Gestational age, weeks		
28–34	26 (9.3)	<0.001
34–37	78 (27.9)	
>37	176 (62.9)	
Birth weight, g, *n* (%)		
<1,500	4 (1.4)	<0.001
1,501–2,500	96 (34.3)	
>2,501	180 (64.3)	
Admission diagnosis		
Neonatal jaundice	106 (37.9)	<0.001
Neonatal infectious pneumonia	102 (36.4)	
Neonatal hypoxic–ischemic encephalopathy	30 (10.7)	
Premature infants	24 (8.6)	
Neonatal respiratory distress syndrome	14 (5.0)	
Neonatal asphyxia	4 (1.4)	

Comparisons between groups were performed using the one-way ANOVA.

### The most and least important needs of grandparents

The 10 most and least important aspects, based on the results from the NICU-FNI scale, can be found in Table 3. Among the top 10 most important needs, six items for Assurance emerged as a significant factor, with grandparents expressing a strong desire for their infant to receive the best care even in their absence from the hospital. Additionally, grandparents emphasized the importance of being informed about their baby’s condition and treatments, having their questions answered honestly, and receiving explanations in a clear and understandable manner. The need for Proximity and Information regarding the baby’s progress also ranked high. On the other hand, among the 10 least important needs, aspects related to Comfort and Support were prominent. Specific requirements, such as restroom availability in the waiting room and private areas in the hospital, were ranked lower in importance. Overall, the findings highlight the crucial role of Assurance, Information, and Support in addressing the needs of grandparents in the NICU while suggesting that certain Comfort-related factors may be of lesser priority.

**Table 3:** Ten most and least important needs of grandparents according to NICU-FNI scale scores (*n* = 280).

Ten most important needs of grandparents	NICU-FNI subscale	Mean score (x ± SD)
To be assured that the best care possible is being given to my infant even in my absence from the hospital	Assurance	4.28 ± 0.54
To be assured that the best care possible is being given to my infant	Assurance	4.25 ± 0.52
To know the expected outcome for my infant	Assurance	4.24 ± 0.52
To have my questions regarding my infant answered honestly	Assurance	4.23 ± 0.48
To have the medical staff provide an explanation in simple terms	Assurance	4.21 ± 0.54
To know how my infant is being treated medically	Information	4.21 ± 0.51
To know specific facts concerning my infant’s progress	Proximity	4.18 ± 0.55
To know the expected outcome for my infant	Assurance	4.17 ± 0.57
To know the expected outcome	Information	4.14 ± 0.45
To have explanations of the NICU environment before entering for the first time	Support	4.14 ± 0.55
Ten least important needs of grandparents		
To have a bathroom near the waiting room	Comfort	2.87 ± 1.04
To have a place to be alone while in the hospital	Support	2.86 ± 0.94
To have friends/family nearby	Support	2.84 ± 1.14
To have classes about premature infant’s special care needs	Information	2.83 ± 1.02
To be able to talk to other parents whose infant is in the neonatal intensive care unit or has had a similar situation	Support	2.83 ± 0.87
To have a support group of other families available	Support	2.79 ± 0.94
To be allowed to have my infant’s siblings visit	Support	2.76 ± 0.94
To have friends/family nearby	Support	2.75 ± 1
To have someone to be concerned with my health	Support	2.68 ± 1.05
To have a telephone in the waiting room	Comfort	2.13 ± 0.89

The Neonatal Intensive Care Unit Family Need Inventory Scale is categorized into five dimensions: Assurance, Information, Proximity, Support, and Comfort. NICU, neonatal intensive care unit; NICU-FNI, NICU-Family Need Inventory.

### NFNI subscale scores and their rankings

Among the identified subscales, “Assurance” received the highest mean score of 4.07 ± 0.49, ranking it as the most important need for grandparents in the NICU setting. This underscored the significance of providing reassurance and emotional Support to grandparents during this difficult time. The subscales “Information” and “Proximity” obtained identical mean scores of 3.50 ± 0.47 and 3.50 ± 0.46, respectively, indicating their equal importance in the second and third positions. These findings highlight the value of clear and comprehensive Information about the infant’s condition and the opportunity for grandparents to be physically close to their grandchild. The “Support” subscale had a mean score of 3.10 ± 0.39, placing it fourth in importance. This suggests that grandparents greatly benefit from various forms of Support, such as emotional, practical, and social support, while their grandchild is in the NICU. Finally, the “Comfort” subscale received the lowest mean score of 2.90 ± 0.53; it ranked fifth, indicating that although Comfort is still important, it may be less prioritized by grandparents than other needs. In conclusion, grandparents in the NICU setting primarily value Assurance, followed by the need for Information and Proximity. Support is also crucial, whereas Comfort holds a slightly lower priority. These findings shed light on the needs of grandparents and emphasize the importance of addressing them to provide optimal Support and care during their grandchild’s NICU stay (Table 4).

**Table 4:** Mean importance of neonatal family needs inventory subscales for grandparents.

NICU-FNI subscale	Mean score (x ± SD)	Ranking of important needs
Assurance	4.07 ± 0.49	1
Information	3.50 ± 0.47	2
Proximity	3.50 ± 0.46	3
Support	3.10 ± 0.39	4
Comfort	2.90 ± 0.53	5

NICU, neonatal intensive care unit; NICU-FNI, NICU-Family Need Inventory.

### Correlation between grandparents’ needs and demographic characteristics

In Table 5, we identify statistically significant correlations between the needs of grandparents and their demographic attributes, as well as those of preterm infants. Specifically, the age of the grandparents is positively associated with the need for Proximity (*r* = 0.143; *p* < 0.05), suggesting older grandparents have more demand for proximity to preterm infants. The educational attainment of grandparents is positively related to the need for Information (*r* = 0.135; *p* < 0.05), yet inversely related to the need for Proximity (*r* = 0.159; *p* < 0.01), revealing that grandparents with lower education levels may seek less Information but desire greater Proximity to their grandchildren. The employment status of grandparents is associated with Assurance (*r* = 0.122; *p* < 0.05), Proximity (*r* = 0.219; *p* < 0.01), and Support (*r* = 0.138; *p* < 0.05), suggesting that retired grandparents may seek a more profound connection with grandchildren. The place of residence of grandparents is positively associated with the need for Assurance (*r* = 0.179; *p* < 0.01), Proximity (*r* = 0.168; *p* < 0.01), and Comfort (*r* = 0.118; *p* < 0.05), indicating that grandparents residing in rural areas may exhibit a greater need for these aspects than those in urban settings.

**Table 5:** Statistically significant correlations between grandparents’ needs and demographic characteristics.

Characteristics	Assurance	Information	Proximity	Support	Comfort	Mean score
Sex	−0.026	−0.019	−0.081	−0.06	−0.032	−0.058
Age	−0.034	−0.046	0.143*	0.045	−0.036	0.015
Education level	−0.096	0.135*	−0.159**	−0.002	−0.028	−0.042
Employment status	0.122*	0.087	0.219**	0.138*	0.084	0.174**
Place of residence	0.179**	0.05	0.168**	−0.007	0.118*	0.144*
Gestational age	−0.394**	−0.300**	−0.098	−0.157**	−0.242**	−0.330**
Birth weight	−0.263**	−0.214**	0.013	−0.018	−0.082	−0.159**

Values represent the Pearson’s correlation coefficient; “*” means *p* < 0.05, “**” means *p* < 0.01. Abbreviations: NICU, neonatal intensive care unit; NICU-FNI, NICU-Family Need Inventory.

Regarding the attributes of preterm infants, there is a negative correlation between gestational age and the grandparents’ needs for Assurance, Information, Support, and Comfort, as well as the overall need score. This indicates that as the gestational age of a premature infant at birth increases, the needs of the grandparents decrease. The birth weight is inversely related to the need for Assurance (*r* = −0.263; *p* < 0.01) and Information (*r* = −0.214; *p* < 0.01), potentially indicating heightened concern among grandparents for the health issues pertaining to infants with lower birth weights.

### Multiple linear regression analysis of needs among grandparents

Multiple linear regression analysis was employed to elucidate the determinants of grandparents’ needs. Six family needs models were identified for the analysis (covering the grandparents’ needs), focusing on the mean scores of NICU-FNI, including the needs for Assurance, Information, Proximity, Support, and Comfort.

The results from the multiple regression models, which analyzed the needs of grandparents across different dimensions, identify key predictors that are associated with various aspects of Support in the NICU as depicted in Table 6. The mean score model highlights significant associations between the mean score and employment status (*p* = 0), place of residence (*p* = 0.038), and birth weight (*p* = 0.001). This indicates that grandparents who are retired, live in rural areas, and have grandchildren with lower birth weights have greater needs. In the domain of Assurance, the predictors of significance were employment status (*p* = 0.005), place of residence (*p* = 0.027), and gestational age (*p* = 0), indicating that grandparents who are retired and residing in rural locales, along with their grandchildren of lower birth weights, possess an augmented need for Assurance. The Information model revealed that the level of education (*p* = 0.006) and gestational age (*p* = 0) exert significant impacts on the need for Information, indicating that grandparents possessing higher educational qualifications and their grandchildren with reduced birth weights exhibit a more substantial need for Information. The Proximity model identified employment status (*p* = 0.002) and place of residence (*p* = 0.008) as significant predictors, suggesting that grandparents who are retired and reside in rural areas have an increased need for Proximity. In the Comfort model, gestational age (*p* = 0) was the only factor identified as a significant predictor, whereas the Support model showed no significant predictors. The findings highlight the importance of considering specific factors when addressing the diverse needs of grandparents in the NICU, providing valuable insights for tailored Support interventions.

**Table 6:** Multiple regression model explaining grandparents’ needs.

	Unstandardized coefficients		Standardized coefficients	*t*	*p*	VIF
	*B*	standard error	Beta			
The model that explains grandparents’ needs for mean score						
(Constant)	3.355	0.134		24.964	0	
Employment status	0.059	0.017	0.194	3.533	0	1.02
Place of residence	0.081	0.039	0.117	2.087	0.038	1.075
Gestational age	−0.295	0.047	−0.564	−6.215	0	2.798
Birth weight	0.2	0.062	0.297	3.211	0.001	2.912
The model that explains grandparents’ needs for Assurance						
(Constant)	4.273	0.189	–	22.633	0	–
Employment status	0.067	0.024	0.156	2.861	0.005	1.02
Place of residence	0.122	0.055	0.124	2.227	0.027	1.075
Gestational age	−0.377	0.067	−0.51	−5.654	0	2.798
Birth weight	0.149	0.088	0.156	1.698	0.091	2.912
The model that explains grandparents’ needs for Information						
(Constant)	4.164	0.155		26.781	0	
Education level	−0.07	0.025	−0.159	−2.796	0.006	1.006
Gestational age	−0.27	0.068	−0.379	−3.994	0	2.811
Birth weight	0.077	0.087	0.084	0.891	0.374	2.8
The model that explains grandparents’ needs for Proximity						
(Constant)	2.9	0.121		24.067	0	
Age group	0.025	0.027	0.059	0.921	0.358	1.234
Education level	0.04	0.026	0.093	1.545	0.124	1.102
Employment status	0.079	0.026	0.192	3.061	0.002	1.197
Place of residence	0.147	0.055	0.159	2.666	0.008	1.071
The model that explains grandparents’ needs for Support						
(Constant)	3.342	0.112		29.941	0	
Education level	−0.003	0.022	−0.009	−0.145	0.885	1.005
Gestational age	−0.093	0.035	−0.157	−2.648	0.009	1.005
The model that explains grandparents’ needs for Comfort						
(Constant)	3.246	0.174		18.624	0	
Place of residence	0.078	0.064	0.072	1.219	0.224	1.042
Gestational age	−0.184	0.048	−0.228	−3.838	0	1.042

## Discussion

The care provided in the neonatal intensive care unit (NICU) is vital for preterm infants (Cao et al., 2021). However, this type of care presents significant economic, psychological, and health challenges for the families of preterm infants (Geng et al., 2022; Grunberg et al., 2022). Despite ample evidence regarding the needs of parents in the NICU, the needs of other family members, such as grandparents, are often overlooked (Adama et al., 2022; Maleki et al., 2022). Grandparents, however, play a crucial role in providing support to the family (Hillis et al., 2021), and it is important to understand their needs in order to improve NICU care practices. This study aimed to provide a comprehensive investigation of the needs of this particular group, thereby making an additional contribution to the existing body of literature.

The findings of this study reveal that grandparents’ foremost concern is the Assurance of the infant’s safety and wellbeing. Studies involving parents of preterm infants in the NICU have consistently identified Assurance as the top priority (Alsaiari et al., 2019; Govindaswamy et al., 2020). Similarly, family members of critical care patients have emphasized the importance of Assurance (Kang et al., 2020), and nurses have also deemed Assurance as the most critical need for family members of patients admitted to critical care units (Khatri Chhetri and Thulung, 2018). It is both expected and understandable that all family members, including grandparents and parents, prioritize health assurance for these infants, recognizing it as a crucial necessity.

In the hierarchy of importance among the remaining four dimensions, the needs of grandparents of preterm infants in the NICU are prioritized as follows: Information, Proximity, Support, and Comfort. In comparison, the needs of Saudi parents of preterm infants are ranked as Proximity, Information, Comfort, and Support (Alsaiari et al., 2019), while another study focused on the needs of fathers, ranking them as Proximity, Information, Support, and Comfort (Govindaswamy et al., 2020). The variations in family member inclusion across research studies and differences in hospital policies regarding visitation for preterm infants in the NICU may elucidate this discrepancy. In Saudi hospitals, parents are permitted to visit their infants around the clock; thus, Alsaiari et al. excluded two Proximity-related items from the NICU-FNI scale: “to be able to visit my infant at any time” and “to see my infant frequently” (Alsaiari et al., 2019). In China, NICUs employ a closed management approach alongside the traditional practice of “sitting the month.” This results in minimal opportunities for mothers of premature infants to observe their newborns after birth, and even fewer opportunities for other relatives to have contact with the baby at that time (Ling et al., 2022). Consequently, our research emphasizes the heightened significance of Information and Proximity for grandparents. Prior research suggests that the Information needs of family members of patients in critical care are the second most significant, irrespective of the family’s educational or cultural background (Kang et al., 2020; Wesson, 1997). Studies indicate that family members of patients admitted to critical care units frequently seek Proximity and Information as coping mechanisms to alleviate anxiety (Baharoon et al., 2017; Wang et al., 2018). This aligns with the family resilience framework, which underscores the importance of clear communication and emotional closeness as factors that bolster resilience. When family members are well-informed and have opportunities for meaningful involvement, they are better equipped to manage the emotional and psychological stress associated with NICU experiences (Walsh, 2016).

The results of this study reveal a significant negative correlation between the gestational age of preterm infants and the grandparents’ need for Assurance, Information, Support, and Comfort, as well as the overall need score. This suggests that as the gestational age of a premature infant at birth increases, the needs of the grandparents decrease. It is worth noting that previous studies have indicated a similar trend: as an infant spends more time in the NICU, family members place less emphasis on seeking Information about the infant (Nicholas, 2018; Alsaiari et al., 2019). Furthermore, the psychological stress, anxiety, and fear experienced by family members are found to escalate as the infant’s condition deteriorates. However, when there is an improvement in the infant’s health, these negative emotions gradually subside (Heidari et al., 2012; Jackson et al., 2003). This inverse relationship between the severity of the infant’s condition and the emotional state of the family members could be attributed to the fact that the occurrence and severity of multiple diseases are linked to gestational age and birth weight. Conditions such as respiratory distress syndrome (Mikolajcikova et al., 2022), bronchopulmonary dysplasia (Trembath and Laughon, 2012), and necrotizing enterocolitis (Battersby et al., 2017) are more likely to occur in infants with lower gestational age and birth weight.

The needs related to Support (interpersonal and emotional) and Comfort (personal and physical) are the lowest priority for grandparents. Concerning the hospital infrastructure, elements such as the Proximity of a bathroom to the waiting area, the provision of a solitary space within the hospital, and the presence of a phone in the parental waiting room are not the foremost considerations of grandparents. This underscores grandparents’ tendency to place the Comfort and Support needs of the mother and baby above their personal needs, a perspective that aligns with findings on paternal needs (Ireland et al., 2016).

This study does not negate the fact that parental involvement in newborn care within the NICU is a currently favored healthcare approach (Zanoni et al., 2021). Furthermore, decisions regarding emergency treatment for NICU patients and communication with doctors position parents as taking a leading position among family members (Govindaswamy et al., 2020). However, upon the admission of their newborn to the NICU, parents often experience anxiety, depression, guilt, and helplessness due to their unpreparedness and lack of parenting experience, the unpredictability associated with their preterm infant’s stay in an unfamiliar NICU environment, the closed management system of the NICU, the uncertainty of their infant’s medical condition, prognostic worries, and substantial hospital expenses (Diffin et al., 2016; Whittingham et al., 2014). These challenges often result in their inability to understand nurses’ explanations or engage in the caregiving process (Ladani et al., 2017). Moreover, fathers particularly wish for grandparents to assist in alleviating their stress, as mothers require care following childbirth, thereby positioning grandparents as their most trusted allies (Aliabadi et al., 2014). Hence, grandparents could serve as a vital communication bridge between nurses and parents, lowering the communication burden on nurses and fostering a collaborative relationship, ultimately contributing to improved care and expectations for the newborn (Wigert et al., 2013).

As with any survey-based research, our study has certain limitations. First, because of the cross-sectional design of this study, we are unable to assess changes in the needs of grandparents of preterm infants in the NICU over time. Additionally, our sample was limited to grandparents from a specific region in China, which may limit the broader applicability of our findings. Third, the self-completion nature of the questionnaires introduces the potential for bias due to social desirability. Given these factors, it is important to interpret the research findings with caution.

## Conclusion

This study aimed to broaden the scope of research concerning the needs of grandparents with preterm infants in NICU, complementing the existing studies that have predominantly centered on the needs of parents. The research reveals that grandparents primarily desire Assurance regarding the safety and health of their grandchild, indicating a substantial level of concern about the quality of care. Additionally, secondary needs related to Information and Proximity were identified.

The findings demonstrate a significant negative correlation between the gestational age of preterm infants and the grandparents’ needs for Assurance, Information, Support, and Comfort. As the gestational age increases, the expressed needs of grandparents tend to diminish, supporting previous findings that suggest family members experience less anxiety and a decreased demand for information as infants stabilize.

The results underscore the critical role grandparents play within the family support system and highlight the necessity of recognizing and addressing their needs in clinical practice. Recognizing these needs is essential not only for alleviating family stress but also for enhancing overall care quality within NICUs. By emphasizing family-centered care, the study advocates for policies and practices that acknowledge and support the involvement of grandparents during such challenging times.

While this research provides valuable insights, its cross-sectional design constrains the ability to monitor changes in needs over time. Moreover, the sample was limited to a specific region in China, potentially affecting the generalizability of the findings. Future research should employ longitudinal designs to assess the evolution of grandparents’ needs and to explore these needs across diverse cultural and healthcare contexts.

In conclusion, this study serves as a call to action for healthcare providers and policymakers to integrate the needs of grandparents into NICU care practices, ensuring they receive appropriate support, communication, and engagement during challenging periods for the entire family. This approach could enhance the psychological wellbeing of families and improve the quality of care in NICUs.

## Data Availability

The original contributions presented in the study are included in the article/Supplementary material, further inquiries can be directed to the corresponding author.
