# Supplementary material for: Assessing the needs of grandparents of preterm infants in neonatal intensive care units: a cross-sectional study
Source: Front Psychol. 2024 Nov 19;15:1433391. doi: 10.3389/fpsyg.2024.1433391 (PMC11613964; doi:10.3389/fpsyg.2024.1433391)
Supplement: Supplementary file 1 [file Table_1.DOCX]

# Survey on needs in Grandparents of preterm infants in Neonatal Intensive care units

## Part A: General characteristics

We appreciate your participation! This brief survey is anonymous and will not be linked to your work record. Please refer to the basic information and chooses the best option from among multiple choice.

| **General characteristics of Grandparents** | |
| --- | --- |
| 1. Gender | Male □; Female □ |
| 2. Age | _______years |
| 1. Place of residence | Town/city □; Country side □ |
| 4. Education level | College and above □; Junior high school/technical secondary school □; Junior middle school □; Primary school and below □ |
| 5. Employment Status | Employed full-time □; Employed part-time □; Unemployed □; Retired □ |
| **General characteristics of the newborn (Nurses assist grandparents in filling out forms based on patients' medical records)** | |
| Gestational age | 28–34 weeks □; 34–37 weeks □; >37 weeks □ |
| Birthweight | <1500g □; 1501–2500g □; >2501g □ |
| Admission diagnosis | Neonatal jaundice □; Neonatal infectious pneumonia □; Neonatal hypoxic-ischemic encephalopathy  Premature infants □; Neonatal respiratory distress syndrome □; Neonatal asphyxia □; Others □ |

## Part B: The Neonatal Intensive Care Unit Family Need Inventory Scale

| Kindly mark the importance level of the following items based on your needs (Select one option per row). | | | | | | |
| --- | --- | --- | --- | --- | --- | --- |
| No. | Items | extremely important | important | moderately important | unimportant | extremely unimportant |
| 1 | To know the expected outcome for my infant | □ | □ | □ | □ | □ |
| 2 | To have explanations of the NICU environment before entering for the first time | □ | □ | □ | □ | □ |
| 3 | To be able to visit at any time | □ | □ | □ | □ | □ |
| 4 | To be able to communicate with my infant's doctor every day | □ | □ | □ | □ | □ |
| 5 | To have questions about my infant answered honestly | □ | □ | □ | □ | □ |
| 6 | To have friends/family nearby | □ | □ | □ | □ | □ |
| 7 | To have comfortable furniture in the waiting room | □ | □ | □ | □ | □ |
| 8 | To know which medical staff can provide information about my infant's health condition | □ | □ | □ | □ | □ |
| 9 | To be able to communicate my emotions with the medical staff | □ | □ | □ | □ | □ |
| 10 | To be assured that the best care possible is being given to my infant | □ | □ | □ | □ | □ |
| 11 | To have family assistance close by | □ | □ | □ | □ | □ |
| 12 | To have classes about premature infants special care needs | □ | □ | □ | □ | □ |
| 13 | To have a support group of other families available | □ | □ | □ | □ | □ |
| 14 | To have another person with me when visiting the unit | □ | □ | □ | □ | □ |
| 15 | To know why things were done for my infant | □ | □ | □ | □ | □ |
| 16 | To be assured that the best care possible is being given to my infant even in my absence from the hospital | □ | □ | □ | □ | □ |
| 17 | To have a telephone in the waiting room | □ | □ | □ | □ | □ |
| 18 | To have comfortable furniture in the waiting room | □ | □ | □ | □ | □ |
| 19 | To have a pastor,clergy,or other person from my church visit | □ | □ | □ | □ | □ |
| 20 | To have targeted informational support regarding my infant-related issues | □ | □ | □ | □ | □ |
| 21 | To have someone to be concerned with my health | □ | □ | □ | □ | □ |
| 22 | To be able to communicate my infant's condition with a fixed nurse | □ | □ | □ | □ | □ |
| 23 | To have a bathroom near the waiting room | □ | □ | □ | □ | □ |
| 24 | To be able to communicate the worst possible condition of my infant with medical staff | □ | □ | □ | □ | □ |
| 25 | To have someone be concerned with my health. | □ | □ | □ | □ | □ |
| 26 | To have explanation to me in simple terms by medical staff | □ | □ | □ | □ | □ |
| 27 | To have a bathroom near the waiting room | □ | □ | □ | □ | □ |
| 28 | To be called at home about important changes in my infant’s condition | □ | □ | □ | □ | □ |
| 29 | To receive information about infant at least once a day | □ | □ | □ | □ | □ |
| 30 | To see my infant frequently | □ | □ | □ | □ | □ |
| 31 | To know specific facts concerning my infant’s progress | □ | □ | □ | □ | □ |
| 32 | To feel that the hospital personnel care about my infant. | □ | □ | □ | □ | □ |
| 33 | To have a support group of other families available | □ | □ | □ | □ | □ |
| 34 | To be able to talk to other parents whose infant is in the neonatal intensive care unit or has had a similar situation | □ | □ | □ | □ | □ |
| 35 | To be allowed to have my infant’s siblings visit | □ | □ | □ | □ | □ |
| 36 | To know the expected outcome | □ | □ | □ | □ | □ |
| 37 | To know specific facts concerning my infant's progress | □ | □ | □ | □ | □ |
| 38 | To know the expected outcome for my infant | □ | □ | □ | □ | □ |
| 39 | To have a place to be alone while in the hospital | □ | □ | □ | □ | □ |
| 40 | To receive help in responding to the reactions of my infant's siblings | □ | □ | □ | □ | □ |
